# Warmth and competence perceptions of key protagonists are associated with containment measures during the COVID-19 pandemic: Evidence from 35 countries

**DOI:** 10.1038/s41598-022-25228-9

**Published:** 2022-12-08

**Authors:** Maria-Therese Friehs, Patrick F. Kotzur, Christine Kraus, Moritz Schemmerling, Jessica A. Herzig, Adrian Stanciu, Sebastian Dilly, Lisa Hellert, Doreen Hübner, Anja Rückwardt, Veruschka Ulizcay, Oliver Christ, Marco Brambilla, Jonas De keersmaecker, Federica Durante, Jessica Gale, Dmitry Grigoryev, Eric R. Igou, Nino Javakhishvili, Doris Kienmoser, Gandalf Nicolas, Julian Oldmeadow, Odile Rohmer, Bjørn Sætrevik, Julien Barbedor, Franco Bastias, Sebastian B. Bjørkheim, Aidos Bolatov, Nazire Duran, Andrej Findor, Friedrich Götz, Sylvie Graf, Anna Hakobjanyan, Georgios Halkias, Camellia Hancheva, Martina Hřebíčková, Matej Hruška, Shenel Husnu, Kamoliddin Kadirov, Narine Khachatryan, Francisco G. Macedo, Ana Makashvili, Maylin Martínez-Muñoz, Eric Mercadante, Luiza Mesesan Schmitz, Andreas Michael, Nozima Mullabaeva, Félix Neto, Joana Neto, Merve Ozturk, Svitlana Paschenko, Agnieszka Pietraszkiewicz, Charis Psaltis, Yuting Qiu, Mirjana Rupar, Adil Samekin, Katharina Schmid, Sabine Sczesny, Yiwen Sun, Annika M. Svedholm-Häkkinen, Aleksandra Szymkow, Enoch Teye-Kwadjo, Claudio V. Torres, Luc Vieira, Illia Yahiiaiev, Vincent Yzerbyt

**Affiliations:** 1 FernUniversität in Hagen, Hagen, Germany; 2 Department of Psychology, Durham University, South Road, Durham, DH1 3LE UK; 3 GESIS Leibniz-Institut für Sozialwissenschaften, Mannheim, Germany; 4 University of Milano-Bicocca, Milan, Italy; 5 Esade, Ramon Llull University, Barcelona, Spain; 6 University of Canterbury, Christchurch, New Zealand; 7 HSE University, Moscow, Russia; 8 University of Limerick, Limerick, Ireland; 9 Ilia State University, Tbilisi, Georgia; 10 Rutgers University, New Brunswick, USA; 11 Swinburne University of Technology, Melbourne, Australia; 12 University of Strasbourg, Strasbourg, France; 13 University of Bergen, Bergen, Norway; 14 Université Catholique de Louvain, Louvain-la-Neuve, Belgium; 15 Universidad Católica de Cuyo/National Scientific and Technical Research Council, San Juan, Argentina; 16 Astana Medical University, Astana, Kazakhstan; 17 Comenius University in Bratislava, Bratislava, Slovakia; 18 The University of British Columbia, Vancouver, Canada; 19 The Czech Academy of Sciences, Prague, Czechia; 20 Yerevan State University, Yerevan, Armenia; 21 Copenhagen Business School, Frederiksberg, Denmark; 22 Sofia University “St. Kliment Ohridski”, Sofia, Bulgaria; 23 Eastern Mediterranean University, Famagusta, Cyprus; 24 University of Innovative and Social Economics, Tashkent, Uzbekistan; 25 University of Brasilia, Brasília, Brazil; 26 Transilvania University of Brasov, Brașov, Romania; 27 University of Cyprus, Nicosia, Cyprus; 28 National University of Uzbekistan, Tashkent, Uzbekistan; 29 University of Porto, Porto, Portugal; 30 Universidade Portucalense, Porto, Portugal; 31 Taras Shevchenko National University of Kyiv, Kyiv, Ukraine; 32 University of Bern, Bern, Switzerland; 33 M. Narikbayev KAZGUU University, Astana, Kazakhstan; 34 Tampere University, Tampere, Finland; 35 SWPS University of Social Sciences and Humanities, Warsaw, Poland; 36 University of Ghana, Accra, Ghana; 37 Jagiellonian University, Krakow, Poland

**Keywords:** Human behaviour, Lifestyle modification

## Abstract

It is crucial to understand why people comply with measures to contain viruses and their effects during pandemics. We provide evidence from 35 countries (*N*_total_ = 12,553) from 6 continents during the COVID-19 pandemic (between 2021 and 2022) obtained via cross-sectional surveys that the social perception of key protagonists on two basic dimensions—warmth and competence—plays a crucial role in shaping pandemic-related behaviors. Firstly, when asked in an open question format, heads of state, physicians, and protest movements were universally identified as key protagonists across countries. Secondly, multiple-group confirmatory factor analyses revealed that warmth and competence perceptions of these and other protagonists differed significantly within and between countries. Thirdly, internal meta-analyses showed that warmth and competence perceptions of heads of state, physicians, and protest movements were associated with support and opposition intentions, containment and prevention behaviors, as well as vaccination uptake. Our results have important implications for designing effective interventions to motivate desirable health outcomes and coping with future health crises and other global challenges.

## Introduction

Pandemics are large-scale outbreaks of infectious diseases that have major detrimental consequences for many people around the globe, with the potential to cause social, political, and cultural changes^1^. The COVID-19 pandemic triggered by a novel coronavirus discovered in December 2019 was no different: Having spread globally in a matter of weeks, numerous counter-measures were imposed, including vaccination campaigns once suitable vaccines were available^2^. Additionally, and importantly, previously unfamiliar individuals, groups, social movements, and organizations arose in public discourses regarding COVID-19^3,4^, creating new divides in many contemporary societies—between those who supported and those who opposed COVID-19 prevention measures^5,6^. Understanding why people approve of, reject, or comply with measures aimed at reducing the spread of contagious viruses or the severity of infections is important for researchers, policymakers, and those who instantiate such measures. Such an understanding could help us to effectively cope with future pandemics and serve as a starting point to design effective interventions to motivate desirable health outcomes.

Previous studies suggest a number of factors that influence the degree to which people in different societies support or reject COVID-19 pandemic-related health behaviors, such as self-interest, pro-sociality, religiosity, conspiracy beliefs, trust, national identification, perceived effectiveness, and quality of institutions^7–14^. In this paper, we add to this literature by arguing that to understand why people support or oppose certain measures, it is crucial to understand: (I) which individuals, groups, social movements, or organizations (referred to as ‘protagonists’ in the following) people identify as relevant, and (II) how people perceive these protagonists. Indeed, previous research suggests that social perceptions of protagonists are related to containment behaviors: For instance, more benevolent social perceptions of the elderly, as vulnerable protagonists who were disproportionately affected by the pandemic, were associated with more containment behaviors in Italy during the COVID-19 pandemic^15^. The less benevolent the perception of Chinese people (protagonists perceived to be partially responsible for the outbreak of the virus) during the pandemic, the more the endorsement of restrictive policies in a UK sample^16^. Lastly, it has been suggested that trust in institutions, such as governments and health experts, positively predicts compliance with measures recommended by these institutions^17–19^.

Despite these first indications about the relationship between the social perception of protagonists and preventive behaviors, little is known about which protagonists are particularly salient in the COVID-19 pandemic, how they are generally evaluated, and how these perceptions relate to pandemic-related behaviors. This knowledge would provide a valuable sense of whose statements and actions people deem most important in the pandemic, and how their evaluations are reflected in the adherence of pandemic-related behaviors. The present study addresses this gap by investigating societally shared perceptions of relevant protagonists in the COVID-19 pandemic and their correlates with pandemic-related behaviors in 35 countries across six continents.

We base our study on the stereotype content model (SCM)^20^ and its extension^21^, which provide a well-established paradigm to investigate societally shared perceptions^22^. Originally, the SCM was designed to shed light on societally shared perceptions of social groups in general^20^, and has been adapted to investigate domain-specific social perceptions, including migrants^23,24^, occupations^25,26^, or institutions and brands^27^. Recently, the model has been found to be compatible with other prominent models of social perceptions^22^.

The SCM^20^ suggests that people make sense of the social world by addressing two fundamental questions when evaluating protagonists: Are their intentions generally perceived as friendly or hostile? And are they capable of enacting their (friendly or hostile) intentions? These evaluations translate into warmth and competence perceptions, respectively, on which the social perception of protagonists can vary independently. Decades of research have shown that these two dimensions are fundamental to social perception and jointly predict a wide range of outcomes^28^. Importantly, the model has been extended to suggest that warmth and competence perceptions also engender behavioral intentions toward protagonists^21^: Higher regard on warmth and competence leads to more facilitation (e.g., supporting), whereas lower regard on these dimensions leads to more harm (e.g., ignoring)^29^. (The original studies theorized that warmth and competence are each associated with intentions differing in their intensities and directness^21^. Empirically, both warmth and competence correlate with facilitation and harm intention^23,30^, which is why we propose a more parsimonious approach refraining from such distinctions here).

The model has been designed to predict behavioral intentions towards protagonists, which we also intend to do in this study to gain insights into the relationship between social perceptions and protagonist-specific intentions. Crucially, we also go beyond this model by suggesting that social perceptions of protagonists relevant to the COVID-19 pandemic can also help us to understand more general pandemic-related behaviors. Following Tajfel and Turner’s^31^ seminal work on categorization and identity, we argue that protagonists salient within the pandemic context are associated with specific positions towards pandemic-related health behaviors (e.g., whether they are in favor or against containment behaviors). This position may or may not align with people’s own position on the behaviors (what Tajfel and Turner^31^ called a self-categorization prototype). When these positions align—that is, when protagonists’ positions are perceived to be more compatible with individuals’ own beliefs—people will evaluate protagonists more favorably on stereotype content dimensions^32^. Favorable evaluations of a protagonist who supports a certain health-related behavior may promote the same behavior in the evaluator. This is because people strive to achieve prototypical behavior shown by protagonists who are considered societal and ingroup prototypes (high warmth and high competence groups^20,31^. Indeed, previous research on COVID-19 related behaviors emphasized the special role of trust (e.g.^10^), related to warmth. Moreover, people are more likely to engage in certain behaviors if there is a sense of collective efficacy^33,34^, which we argue is fed by protagonists’ competence perceptions.

For example, heads of state, who are undoubtedly major protagonists actively engaged in managing the pandemic, are generally known to advocate preventive and curative measures^35^. (Notable exceptions were the then-heads of state of the US and Brazil, Trump and Bolsonaro respectively, who downplayed the impact of the virus in the early stages of the pandemic^37^). We argue that the perception of heads of state as well-intentioned (high warmth) and capable (high competence) should correspond with higher adherence to pandemic-related health behaviors propagated by this protagonist. Medical experts, such as physicians, are another example of protagonists actively engaged in managing the pandemic, generally known to advocate pandemic-related health behaviors^36,37^. Similarly, we argue that the social perception of physicians as well-intentioned (high warmth) and capable (high competence) should correspond with higher engagement in pandemic-related health behaviors. Finally, anti-vaccination protest movements across the globe are known to have the prototypical position of being skeptical of pandemic-related behaviors^38^. Accordingly, the perception of anti-vaccination movements as having the best intentions (high warmth) and being capable of carrying out their intentions (high competence) should align with low commitment to pandemic-related behaviors. The attitude–behavior link is strongest when both are on the same level of specificity^39–41^. Accordingly, we expect the associations between the specific perceptions of protagonists and specific behaviors which are targeted at the protagonist in question (e.g., supporting or opposing the protagonist) to be larger than the association with general pandemic-related behavior. As such, we argue and test the assumption that the social perception of relevant protagonists during a pandemic corresponds with the support or opposition to pandemic-related behaviors.

While pursuing these goals, we acknowledge that pandemics, such as the COVID-19 pandemic, are global phenomena. Despite the global reach, decades of cross-cultural psychological research suggest that generalizations from one country context to another are not always warranted^42^. Additionally, responses to the pandemic have significantly varied in terms of nature, promptness, and extent, which does not permit us to generalize from one context to another without empirical validation. To avoid falling prey to potential biases associated with exclusively focusing on selected WEIRD (Western, educated, industrial, rich and democratic) samples^42^, we base our research on 35 countries from six continents that were affected by the pandemic.

To summarize: (I) We identified relevant protagonists who were salient during the COVID-19 pandemic; we investigated how these protagonists were perceived on SCM dimensions warmth and competence—both (II) how their perceptions differed *within* countries, and (III) how the perceptions of selected key protagonists varied *between* countries; finally, (IV) we examined how these perceptions related to pandemic-related behaviors—both more generally, and targeted at specific protagonists. Our pre-registered hypotheses are that high warmth and competence evaluations of protagonists generally in favor of protective measures (e.g., heads of state, physicians) will be associated with support of pandemic-related behaviors, such as engaging in preventive measures or receiving vaccinations, whereas high warmth and competence evaluations of protagonists rejecting protective measures (e.g., protest movements) will be associated with opposition to these measures.

## Results

We present comprehensive and country-specific results in a ShinyApp, https://jaherzig.shinyapps.io/Covid19-Protagonists. To identify protagonists who are perceived as most salient in the COVID-19 pandemic, we asked *N*_total_ = 1016 participants (*n*s = 20–48 per country) in a pilot study to use an open-ended format to nominate protagonists they felt were most relevant in their country^43,44^. The twelve most prominent protagonists for each country are summarized in the ShinyApp, tab “Who are relevant protagonists?” subtab “Protagonist labels”. Total and relative frequencies of these protagonists are graphically and numerically summarized in the subtab “Nominated protagonist categories”. Heads of state, health staff, and protest movements, under which we subsume any locally relevant COVID-19 denial, protest, conspiracy theorist or anti-vaccination movement, were the most prevalent protagonists across all countries. The most common health staff protagonist was physicians, with very few exceptions. We thus use this protagonist label in the following, and highlight in the supplementary materials whenever a different label was used. Other protagonists who were mentioned (in the order of relative frequency) were: protagonists of national and local governments; health experts and scientists; protagonists associated with the ministry of health and representatives; civil protection (military and police); social institutions and volunteer or humanitarian work; media and press; vulnerable groups; protagonists associated with the education or economic sector; politicians in general; and the church.

To investigate the social perception of these protagonists *within* countries in terms of the benevolence of their intentions (warmth) and capability to enact intentions (competence), we asked *N*_total_ = 11,537 participants (*n*s = 205–665 per country) to evaluate the twelve most prominent protagonists of each country on warmth and competence dimensions. These protagonists—with very few exceptions—always included the heads of state, physicians, and a protest movement, as well as nine important country-specific protagonists who emerged from the pilot study and were thus deemed particularly relevant to the respective context. (In China and Uzbekistan, no protest movement was listed as protagonist. In Cyprus (Turkish Cypriot Community), the head of state was omitted, because there was no elected political leader at the time of data collection). We conducted latent mean comparisons of warmth and competence perceptions within countries using the alignment procedure^45^, which are graphically depicted in the tab “How are protagonists perceived within countries?” subtab “Stereotype content model”, and numerically summarized in the subtab “Latent mean values” in the ShinyApp. Analyses, codes and outputs can be found in OSM-3 on the OSF project page. Of note, heads of state overwhelmingly received comparatively negative warmth and competence evaluations (except in China, Germany, Italy, Russia, USA and Uzbekistan). Physicians received comparatively positive evaluations on both warmth and competence dimensions across all countries. Protest movements were consistently negatively evaluated on warmth and competence across all investigated countries.

**Figure 1:**
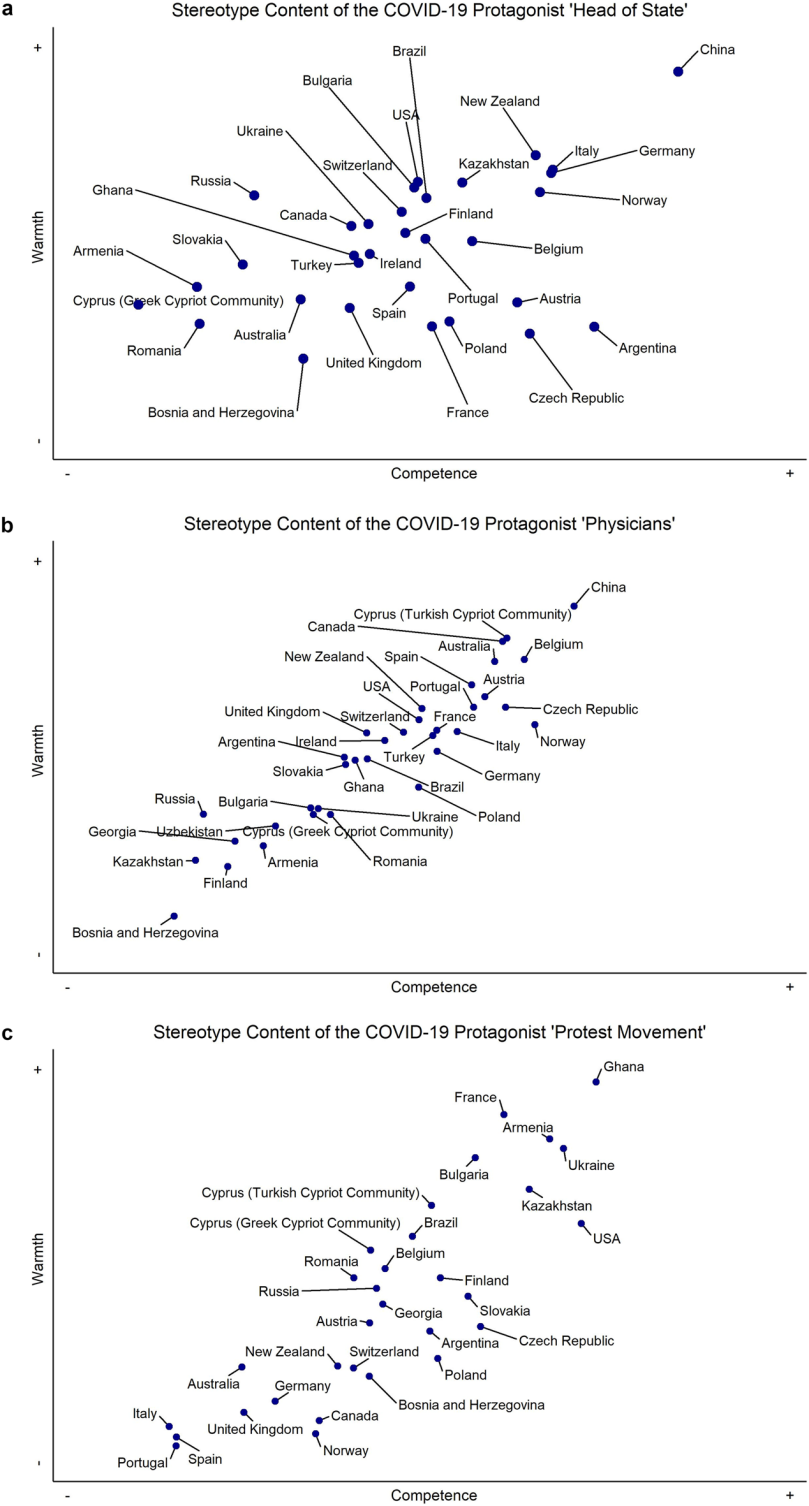
(**a**) The perception of the protagonist ‘heads of state' on warmth and competence dimensions across countries. *Note.* Cyprus (Turkish Cypriot Community) was not included because there was no elected leader at the time of data collection. Georgia and Uzbekistan were not included due to poor model fit. (**b**) The perception of the protagonist 'physicians' on warmth and competence dimensions across countries. (**c**) The perception of the protagonist ‘protest movements' on warmth and competence dimensions across countries. *Note.* Protest movements were not perceived as relevant protagonists in China and Uzbekistan and thus not included in this research. Ireland and Turkey were not included due to poor model fit.

To examine how the social perceptions of heads of state, physicians, and protest movements differed *between* countries, we conducted latent mean comparisons of warmth and competence perceptions for each of the protagonists separately across countries, again using the alignment procedure. We display the results graphically in Fig. 1a–c and in the tab “How are protagonists perceived between countries?” subtab “Stereotype content model”, and numerically in the subtab “Latent mean values” in the ShinyApp. Analyses, codes and outputs can be found in OSM-4. Although heads of state were overall perceived rather negatively *within* countries, they were evaluated most positively in China, New Zealand, Italy, Germany, and Norway, and most negatively in Romania, Armenia, Bosnia-Herzegovina, Slovakia, and Australia. Although physicians were generally among the most highly evaluated groups *within* countries, they were evaluated most positively in China, Cyprus (Turkish Cypriot Community), Belgium, Canada, Australia, Austria, Spain, Portugal, and Czech Republic, most negatively in Bosnia-Herzegovina, Kazakhstan, Finland, Georgia, Russia, Armenia, and Uzbekistan. Finally, although protest movements were overall perceived rather negatively *within* countries, they were evaluated most positively in Ghana, France, Armenia, Ukraine, Bulgaria, and Kazakhstan, and evaluated least positively in Portugal, Italy, Spain, United Kingdom, Germany, and Australia.

Finally, to investigate to what extent social perceptions of the key protagonists heads of state, physicians, and protest movements were linked to pandemic-related health behaviors, we separately correlated warmth and competence perceptions of each of the protagonists with support and opposition measures of the respective protagonist, with the degree to which participants adhered to prevention behaviors, and with vaccination behaviors for each country. We then ran a series of meta-analyses to obtain the overall mean association (*Mr*) between the social perception and each of the behavioral (intention) variables for each of the protagonists separately. Results are summarized in Tables 1, 2 and 3 and in the “How does the social perception of protagonists correlate with pandemic-related behaviors?” Tab of the ShinyApp. Analyses, codes and outputs can be found in OSM-5. As predicted in our pre-registered hypotheses, positive warmth and competence evaluations were related to more support of all protagonists, *Mr*s_Warmth_ = 0.514–0.650, *Mr*s_Competence_ = 0.471–0.591, and less opposition to them, *Mr*s_Warmth_ = − 0.324 to − 0.514, *Mr*s_Competence_ = − 0.281 to − 0.443. Additionally, as predicted, positive warmth and competence evaluations of protagonists in favor of protective measures (heads of state, physicians) were associated with support of pandemic-related behaviors, including engaging in more preventive measures, *Mr*s_Warmth_ = 0.108–0.141, *Mr*s_Competence_ = 0.087–0.142, and receiving a vaccination, *Mr*s_Warmth_ = 0.127–0.162, *Mr*s_Competence_ = 0.103–0.138. In contrast, positive warmth and competence evaluations of the protagonist against protective measures (protest movement) was associated with opposition of the same. As such, positive warmth and competence evaluations were associated with engaging in less preventive measures, *Mr*_Warmth_ = − 0.236, *Mr*_Competence_ = − 0.184, and less intention to get vaccinated, *Mr*_Warmth_ = − 0.315, *Mr*_Competence_ = − 0.264. All meta-analytic relationships were significant with *p*_two-sided_ < 0.001, using both a fixed-effect and random-effect approach. As suggested, associations between warmth and competence perceptions and opposition and support of the protagonists were stronger than those between warmth and competence perceptions and other pandemic-related behaviors, |*Mr*s|= 0.281–0.650 versus |*Mr*s|= 0.087–0.315. Chi-square tests for heterogeneity were significant for all associations, suggesting substantial variability in the size of effects between countries. Some of the variability does not come as a surprise: For instance, whereas most heads of state endorsed preventive measures and vaccinations strongly, Brazil’s then-president Bolsonaro was known for opposing such behaviors^46^. In line with our theorizing, higher warmth and competence perceptions of Bolsonaro were associated with significantly less engagement in preventive measures, and less vaccination behavior in the Brazilian sample.

**Table 1:** Relationships between warmth and competence perceptions of the protagonist ‘heads of state' with pandemic-related behavioral intentions and behaviors within and across 35 countries.

Country	*N*	Support intentions (Heads of State) associated with		Opposition intentions (Heads of State) associated with		Adherence to prevention measures associated with		Vaccination behavior associated with	
		Warmth	Competence	Warmth	Competence	Warmth	Competence	Warmth	Competence
Argentina	273	.644	.589	− .512	− .473	.074	.079	.099	.101
Armenia	286	.424	.369	− .292	− .336	.123	.086	.174	.002
Australia	205	.639	.657	− .191	− .240	− .077	− .072	− .025	− .001
Austria	311	.512	.515	− .170	− .237	− .001	.059	− .004	.081
Belgium	316	.770	.760	− .588	− .577	.298	.323	.388	.358
Bosnia and Herzegovina	293	.280	.349	− .199	− .197	− .031	− .054	− .028	− .096
Brazil	665	.831	.817	− .652	− .641	− .247	− .210	− .109	− .094
Bulgaria	227	.708	.590	− .305	− .340	.238	.295	.306	.182
Canada	366	.494	.627	.026	.088	.055	.038	− .012	− .012
China	255	.547	.522	− .127	− .109	.148	.098	.122	.181
Cyprus (GCC)	285	.604	.602	− .446	− .412	.195	.191	.332	.329
Cyprus (TCC)	*NA*	*NA*	*NA*	*NA*	*NA*	*NA*	*NA*	*NA*	*NA*
Czech Republic	407	.276	.227	− .053	.007	.075	.030	.048	− .002
Finland	293	.820	.811	− .705	− .688	.402	.321	.544	.466
France	287	.802	.682	− .689	− .630	.380	.345	.582	.517
Georgia	286	.654	.606	− .393	− .298	− .043	− .015	− .184	− .229
Germany	351	.650	.525	− .578	− .506	.410	.348	.441	.321
Ghana	309	.580	.525	− .016	.015	.084	.128	.159	.093
Ireland	535	.618	.649	− .076	− .041	.101	.076	.112	.082
Italy	290	.441	.293	− .197	− .151	.001	− .033	.211	.174
Kazakhstan	270	.614	.587	− .177	− .179	.092	.125	− .021	− .069
New Zealand	288	.786	.733	− .464	− .386	.310	.259	.033	.048
Norway	238	.626	.586	− .183	− .122	− .056	− .103	− .048	< .001
Poland	436	.387	.266	− .002	.054	.038	− .016	− .018	.005
Portugal	293	.624	.577	− .433	− .436	.046	.104	.090	.116
Romania	307	.746	.691	− .355	− .225	.138	.091	.090	.115
Russia	325	.780	.682	− .330	− .312	.050	.019	.139	.185
Slovakia	357	.834	.740	− .433	− .386	.288	.284	.405	.383
Spain	327	.650	.527	− .376	− .267	.168	.050	.146	.133
Switzerland	347	.721	.604	− .169	− .003	.186	.184	.254	.120
Turkey	330	.536	.612	− .220	− .225	.146	.154	− .023	.008
United Kingdom	339	.400	.349	− .103	− .026	− .027	− .092	.087	.112
Ukraine	453	.670	.596	− .463	− .382	.040	− .088	− .087	− .066
USA	306	.707	.644	− .132	− .047	.134	.079	.196	.107
Uzbekistan	384	.421	.416	− .309	− .274	.213	.207	.081	.045
**Fixed effects approach**									
M r_z_		.753	.680	− .337	− .289	.109	.087	.128	.104
M r		.637	.591	− .324	− .281	.108	.087	.127	.103
Combined Z		63.434	59.008	− 31.542	− 27.355	11.624	9.533	13.193	10.861
		*p* < .001	*p* < .001	*p* < .001	*p* < .001	*p* < .001	*p* < .001	*p* < .001	*p* < .001
Heterogeneity test (Chi-square)		757.583(33)	631.188(33)	701.020(33)	705.260(33)	277.209(33)	253.605(33)	427.858(33)	322.836(33)
		*p* < .001	*p* < .001	*p* < .001	*p* < .001	*p* < .001	*p* < .001	*p* < .001	*p* < .001
**Random effects approach**									
One-sample t-test		23.302(33)	22.133(33)	− 8.716(33)	− 7.383(33)	4.645(33)	3.941(33)	4.151(33)	3.840(33)
		*p* < .001	*p* < .001	*p* < .001	*p* < .001	*p* < .001	*p* < .001	*p* < .001	*p* < .001

TCC represents Turkish Cypriot Community, GCC represents Greek Cypriot Community. *M r*_*z*_ = weighted mean *r* (Fisher's z transformed). *M r* = weighted mean *r* (converted from *r*_*z*_ to *r*). Small (*r* = .10), medium (*r* = .30), or large (*r* = .50) (cf.^70^).

**Table 2:** Relationships between warmth and competence perceptions of the protagonist ‘physicians' with pandemic-related behavioral intentions and behaviors within and across 35 countries.

Country	*N*	Support intentions (Physicians) associated with		Opposition intentions (Physicians) associated with		Adherence to prevention measures associated with		Vaccination behavior associated with	
		Warmth	Competence	Warmth	Competence	Warmth	Competence	Warmth	Competence
Argentina	273	.460	.409	− .537	− .402	.011	.019	.076	− .044
Armenia	286	.279	.289	− .233	− .189	.135	.145	.100	.077
Australia	205	.709	.501	− .392	− .135	.226	.253	.369	.293
Austria	311	.635	.539	− .562	− .607	.245	.199	.261	.269
Belgium	316	.523	.488	− .262	− .368	.104	.161	.084	.212
Bosnia and Herzegovina	293	.496	.438	− .396	− .385	.072	− .020	.136	.066
Brazil	665	.410	.442	− .281	− .312	.121	.113	.086	.089
Bulgaria	227	.479	.455	− .436	− .365	.192	.240	.171	.093
Canada	366	.420	.384	− .234	− .313	.089	.100	.001	− .098
China	255	.316	.335	− .105	− .073	.096	.111	.054	.096
Cyprus (GCC)	285	.633	.625	− .490	− .488	.241	.267	.481	.521
Cyprus (TCC)	295	.612	.577	− .360	− .390	− .005	.039	.069	.092
Czech Republic	407	.576	.505	− .393	− .294	.093	− .021	.201	.074
Finland	293	.787	.743	− .642	− .644	.304	.274	.445	.457
France	287	.505	.574	− .617	− .676	.178	.303	.351	.397
Georgia	286	.536	.534	− .357	− .329	.304	.321	.074	.011
Germany	351	.379	.385	− .229	− .314	.118	.165	.096	.207
Ghana	309	.366	.365	− .245	− .225	.205	.190	.020	− .020
Ireland	535	.579	.592	− .304	− .315	.183	.204	.163	.108
Italy	290	.090	.006	− .047	− .117	− .055	− .057	.070	.050
Kazakhstan	270	.607	.487	− .194	− .096	.002	.010	− .040	− .137
New Zealand	288	.588	.553	− .564	− .534	.194	.158	.207	.110
Norway	238	.510	.547	− .240	− .351	.021	− .016	.025	.027
Poland	436	.309	.330	.021	− .072	.105	.139	.025	.057
Portugal	293	.569	.541	− .436	− .404	.070	.053	.159	.129
Romania	307	.580	.605	− .396	− .421	.177	.214	.218	.181
Russia	325	.462	.469	− .311	− .329	.161	.141	.121	.143
Slovakia	357	.717	.620	− .497	− .529	.304	.307	.376	.364
Spain	327	.527	.485	− .387	− .487	.126	.128	.305	.259
Switzerland	347	.667	.592	− .258	− .224	.255	.279	.201	.134
Turkey	330	.411	.469	− .167	− .218	.145	.172	.243	.295
United Kingdom	339	.545	.398	− .312	− .230	.016	.023	.166	.114
Ukraine	454	.524	.303	− .286	− .165	.181	.133	.198	.152
USA	306	.449	.310	− .180	− .099	.127	.081	.115	.015
Uzbekistan	383	.440	.452	− .196	− .241	.142	.148	.128	.103
**Fixed effects approach**									
M r_z_		.568	.511	− .337	− .330	.143	.143	.164	.139
M r		.514	.471	− .325	− .319	.142	.142	.162	.138
Combined Z		53.723	49.714	− 34.671	− 34.355	14.923	15.133	17.355	14.783
		*p* < .001	*p* < .001	*p* < .001	*p* < .001	*p* < .001	*p* < .001	*p* < .001	*p* < .001
Heterogeneity test (Chi-square)		378.320(34)	303.594(34)	354.224 (34)	381.468(34)	89.097(34)	115.932(34)	185.677(34)	247.486(34)
		*p* < .001	*p* < .001	*p* < .001	*p* < .001	*p* < .001	*p* < .001	*p* < .001	*p* < .001
**Random effects approach**									
One-sample t-test		21.664(34)	20.852(34)	− 12.487(34)	− 11.918(34)	9.039(34)	8.109(34)	7.672(34)	5.642(34)
		*p* < .001	*p* < .001	*p* < .001	*p* < .001	*p* < .001	*p* < .001	*p* < .001	*p* < .001

TCC represents Turkish Cypriot Community, GCC represents Greek Cypriot Community. *M r*_*z*_ = weighted mean *r* (Fisher's z transformed). *M r* = weighted mean *r* (converted from *r*_*z*_ to *r*). Small (*r* = .10), medium (*r* = .30), or large (*r* = .50) (cf.^70^).

**Table 3:** Relationships between warmth and competence perceptions of the protagonist ‘protest movements' with pandemic-related behavioral intentions and behaviors within and across 35 countries.

Country	N	Support intentions (Protest movements) associated with		Opposition intentions (Protest movements) associated with		Adherence to prevention measures associated with		Vaccination behavior associated with	
		Warmth	Competence	Warmth	Competence	Warmth	Competence	Warmth	Competence
Argentina	273	.738	.670	− .538	− .488	− .188	− .182	− .065	− .084
Armenia	286	.399	.407	− .162	− .123	− .054	.130	− .122	− .055
Australia	205	.663	.492	− .755	− .599	− .244	− .241	− .571	− .392
Austria	311	.774	.777	− .751	− .708	− .169	− .154	− .204	− .256
Belgium	316	.731	.697	− .711	− .683	− .325	− .383	− .544	− .577
Bosnia and Herzegovina	293	.331	.354	− .267	− .348	− .089	− .163	− .293	− .329
Brazil	665	.588	.557	− .471	− .439	− .240	− .219	− .174	− .180
Bulgaria	227	.617	.581	− .703	− .546	− .282	− .106	− .442	− .304
Canada	366	.607	.514	− .572	− .564	− .206	− .209	− .247	− .165
China	*NA*	*NA*	*NA*	*NA*	*NA*	*NA*	*NA*	*NA*	*NA*
Cyprus (GCC)	285	.673	.656	− .596	− .549	− .279	− .249	− .511	− .514
Cyprus (TCC)	295	.668	.586	− .162	− .081	.069	.071	− .103	− .096
Czech Republic	407	.443	.173	− .142	− .087	− .214	− .042	− .141	− .006
Finland	293	.771	.588	− .757	− .556	− .587	− .485	− .662	− .482
France	287	.844	.839	− .835	− .814	− .470	− .512	− .611	− .664
Georgia	286	.497	.168	− .451	− .099	− .199	.025	− .353	− .162
Germany	351	.823	.747	− .697	− .647	− .449	− .390	− .568	− .492
Ghana	309	.598	.501	− .365	− .343	.020	.053	− .181	− .079
Ireland	535	.764	.749	− .566	− .540	− .211	− .216	− .302	− .269
Italy	290	.202	.228	− .199	− .223	.044	− .061	− .077	− .116
Kazakhstan	270	.564	.551	− .510	− .478	− .318	− .250	− .159	− .287
New Zealand	288	.741	.694	− .608	− .593	− .294	− .276	− .297	− .306
Norway	238	.528	.675	− .424	− .396	− .229	− .298	− .231	− .349
Poland	436	.498	.140	− .275	− .090	− .181	.008	− .278	− .049
Portugal	293	.632	.590	− .454	− .485	− .210	− .202	− .120	− .154
Romania	307	.490	.499	− .224	− .185	− .096	− .106	− .075	− .074
Russia	*NA*	*NA*	*NA*	*NA*	*NA*	*NA*	*NA*	*NA*	*NA*
Slovakia	357	.777	.362	− .230	− .270	− .440	− .237	− .574	− .224
Spain	327	.588	.539	− .535	− .428	− .141	− .092	− .282	− .275
Switzerland	347	.751	.365	− .567	− .325	− .333	− .127	− .414	− .215
Turkey	330	.421	.436	− .464	− .448	− .118	− .095	− .266	− .251
United Kingdom	339	.696	.450	− .654	− .419	− .235	− .077	− .237	− .174
Ukraine	454	.731	.798	− .590	− .640	− .286	− .348	− .509	− .578
USA	306	.831	.673	− .646	− .453	− .343	− .224	− .269	− .204
Uzbekistan	*NA*	*NA*	*NA*	*NA*	*NA*	*NA*	*NA*	*NA*	*NA*
**Fixed effects approach**									
M r_z_		.7744	.6287	− .568	− .476	− .241	− .186	− .326	− .270
M r		.650	.557	− .514	− .443	− .236	− .184	− .315	− .264
Combined Z		63.852	54.312	− 50.305	− 43.352	− 23.337	− 18.055	− 31.338	− 26.394
		*p* < .001	*p* < .001	*p* < .001	*p* < .001	*p* < .001	*p* < .001	*p* < .001	*p* < .001
Heterogeneity test (Chi-square)		645.263(31)	804.911(31)	747.565(31)	660.016(31)	239.096(31)	256.159(31)	426.484(31)	405.370(31)
		*p* < .001	*p* < .001	*p* < .001	*p* < .001	*p* < .001	*p* < .001	*p* < .001	*p* < .001
**Random effects approach**									
One-sample t-test		22.711(31)	16.073(31)	− 13.963(31)	− 12.103(31)	− 8.864(31)	− 6.532(31)	− 9.791(31)	− 8.605(31)
		*p* < .001	*p* < .001	*p* < .001	*p* < .001	*p* < .001	*p* < .001	*p* < .001	*p* < .001

TCC represents Turkish Cypriot Community, GCC represents Greek Cypriot Community. *M r*_*z*_ = weighted mean *r* (Fisher's z transformed). *M r* = weighted mean *r* (converted from *r*_*z*_ to *r*). Small (*r* = .10), medium (*r* = .30), or large (*r* = .50) (cf.^70^).

## Discussion

The COVID-19 pandemic brought about significant global changes, including new societal divides between supporters and opposers of pandemic-related measures. To understand why people support or oppose pandemic-related measures, it is important to understand (I) which individuals, groups, social movements, or organizations (‘protagonists’) people perceive as relevant, and (II) how they are perceived. Acknowledging the global reach of the pandemic, we conducted our research in 35 countries to address these questions.

Three protagonists were perceived as particularly relevant in the COVID-19 pandemic—heads of state, physicians, and protest movements. Indeed, in many of the 35 countries included in this study, these three featured heavily in headlines and drove the narratives related to COVID-19. Heads of state (re)presented policies that oftentimes had direct and immediate effect on peoples’ lives. Physicians and medical personnel provided expert advice to navigate this new health crisis and were at the forefront of actively managing it, such as by taking care of those who fell severely ill or by administering vaccines. Protest movements, despite representing a minority opinion in all countries, provided a different perspective to the dominant narrative, and offered alternative ways of thinking about causes, consequences and severity of the pandemic. The fact that the same three protagonists were nominated in most countries with high frequency suggests at least some generalizability of the salience of these protagonists in different parts of the world. These three protagonists represent generalizable categories influencing how people lead their lives, providing leadership (heads of state), expert opinions on ways to overcome the specific challenge (physicians), or opposition to the dominant response (protest movements).

Other protagonists were also frequently perceived as relevant; some who were also trying to actively manage the pandemic (e.g., political entities, such as governments, medical expert advisors, and NGOs); and some who were disproportionately affected by the pandemic (e.g., vulnerable groups, such as elderly and youth). Interestingly, protagonists who were perceived to be partially responsible for the health crisis in other studies (e.g.^16^) were not nominated. Which of these other protagonists emerged as relevant varied across nations—a finding which we were only able to observe thanks to our participant-led nomination approach and coverage of 35 countries.

Investigating how these protagonists were perceived on SCM dimensions provided valuable novel insights, too. Comparing the social perception of protagonists *within* countries revealed that heads of state overwhelmingly received negative warmth and competence evaluations. Participants indicated that they felt heads of state tended to not have their best interests at heart and lacked ability to effectively deal with the pandemic. Overall, other political protagonists beyond heads of state who were nominated as relevant in different countries were also not perceived in a positive light. This observation fits with findings suggesting that politicians are perceived less favorably than other social groups^25,47^. Increasingly, people feel politically alienated in many democratic states^48,49^, and the pandemic has likely exacerbated this trend^50^. Notable exceptions to this overall finding include the countries China, Russia, and Uzbekistan—non-democratic regimes in which participants might have felt compelled to suppress any expression of disapproval, Italy and USA—democratic regimes with a recent change of heads of state, so people might be more willing to give their leadership ‘the benefit of the doubt’—and Germany—a country in which the head of state had accrued considerable popularity beyond party lines over the 16 years in which she was in power^51^. Overall, our results suggest heads of state would do well to project their benevolent intentions and their capability to enact them in the pandemic context and beyond.

Another key observation regarding the social perception of protagonists *within* countries was that physicians received comparatively positive evaluations on both warmth and competence dimensions across all countries. Relatedly, many other health-related protagonists beyond physicians were also evaluated positively on both dimensions. This highly consistent finding across all investigated countries fits well with public displays of the appreciation of health care workers across the globe who were confronted with the herculean task of dealing with millions infected with a new disease^52–55^. A further novel finding was that protest movements were negatively evaluated on warmth and competence across all investigated countries. This is perhaps unsurprising, since the COVID-19 protest movements usually represent a deviant, non-conforming minority threatening the societal group consensus, which is often sanctioned with negative evaluations^56,57^.

Despite the *within*-country trends described above, there was substantial variance in the social perception of the key protagonists *across* countries. Thus, although the trends within countries to appreciate or devalue a group is largely consistent, the extent of these trends varies greatly between them. Our findings provide valuable groundwork for further research investigating why these differences in perceptions across countries may exist. They also provide some initial guidance for multi-lateral organizations working in different countries about which protagonists appear particularly benevolent and capable in the global COVID-19 crisis.

Finally, we examined how the perceptions of the three key protagonists related to pandemic-related behaviors—both generally and targeted at the protagonists themselves. All our pre-registered hypotheses were confirmed. As expected, the higher any protagonist was perceived on warmth and competence, the more participants intended to support them, and the less they intended to oppose them. This finding provides robust support for the basic tenets of the SCM and theoretical extensions we based this research on^20,21^.

Going beyond these predictions and integrating insights of other streams of literature, we are the first to show that high warmth and competence evaluations of protagonists advocating for protective measures (head of state, physicians) were associated with support for health preventative behaviors, such as washing hands, social distancing, and getting vaccinated. High warmth and competence evaluations of protagonists opposed to protective measures (e.g., protest movements) were associated with opposition to the same. As expected, the attitude–behavior link was strongest when both were on the same level of specificity. Accordingly, the associations between the specific perceptions of protagonists and specific behaviors which are targeted at the protagonist in question were larger than the association with general pandemic-related behavior. Nonetheless, this suggests that the side of a societal divide on which people might find themselves at least partially colors the way they tend to view salient protagonists associated with the topic, which in turn is connected to associated behaviors. We believe the finding will be of interest to researchers and policymakers, as our research can guide recommendations on how key protagonists should act in public and serve as a starting point to design effective interventions to motivate desirable societal outcomes—in this health crisis, and similar crises in the future. Such interventions may highlight the good intentions (warmth) and ability (competence) of key protagonists within the respective society that advocate for health preventative behaviors, while undermining the warmth and competence of protagonists that reject such behaviors. This should result in improved health outcomes. These interventions might be inspired by experimental research manipulating warmth and competence perceptions^58^.

Our study has many strengths, including a strong theoretical base on which we built our pre-registered hypotheses; a participant-informed approach; rich data from 35 countries from six continents collected during an ongoing pandemic; and sophisticated statistical modeling and open code that boost confidence in the reliability, validity, reproducibility and replicability of our findings. In addition, we have developed a tool to analyze data structured similar to ours which we share in the supplementary files, as well as a ShinyApp, which helps navigating our rich findings. Nonetheless, there are many ways future research can build on ours.

Our data structure is cross-sectional, which limits our ability to draw causal conclusions when it comes to the link between social perceptions and pandemic-related behaviors. Moreover, alternative explanations of our results are possible. Pandemic-related behavior might be directly influenced by the social perception of key protagonists independently of the regulations that protagonists propagated. We cannot rule out that third variables shown to be associated with containment measures—including self-interest, pro-sociality, religiosity, conspiracy beliefs, trust, national identification, perceived effectiveness, and quality of institutions^7–14^ may independently be associated with the social perception of protagonists and the adherence to containment measures and thus confound the relationship. While most research in the social perception domain is cross-sectional like ours^59^, both theory and empirical work suggest that social perception is likely to engender behavioral intentions and behavior^21,29^. Future research could follow up on ours with longitudinal and experimental designs to show whether this assumption holds in this context as well.

We followed the common practice in the SCM literature to utilize convenience sampling^59^. Early work has shown that warmth and competence evaluations are not dependent on sampling strategy^20^, making convenience sampling a useful and efficient way to obtain data. Future research could attempt to replicate our results with nationally representative samples.

Data collection start and end dates varied by a couple of weeks across countries, which means that the societies from which the data stem were at slightly different stages of the pandemic. Given the nature of the pandemic, where onset, infection rates, and local preventive and curative efforts varied greatly between countries to begin with, as well as different geographic and seasonal characteristics of the included countries, countries would also have been in different stages of the pandemic if start and end dates would have been perfectly aligned. Moreover, the containment measures imposed varied from country to country, sometimes also within country during data collection. Thus, future research could investigate whether the stage of the pandemic might impact results in one way or another.

There is more that future research could explore: We have seen that the social perception of one and the same protagonist can vary significantly between country contexts, reflected in significantly different warmth and competence means across countries for heads of state, physicians, and protest movements. Our results also suggest that there was substantial variance in the relationship between social perception and pandemic-related behavior and behavioral intentions across countries. Future research could explore why that is.

What is more, our research was limited to the investigation of our research questions and predictions in the COVID-19 pandemic context. As such, we encourage research to investigate to what extent our findings might be specific to the present pandemic context or generalize to other global crises. Given the current concerning developments associated with other health crises, such as monkeypox^60^, climate change crises, such as heat waves, droughts, and extreme precipitation^61^, as well as armed conflicts, such as wars^62^, we believe it would be valuable to see to what extent our findings translate to these and other large-scaled challenges as well.

## Conclusion

The COVID-19 pandemic triggered massive changes across the globe. Among them, new individuals, groups, social movements, and organizations arose in public discourses. We have tested and shown for the first time that across 35 countries there are some universals and differences when it comes to which protagonists are perceived as relevant in the pandemic context, as well as how they are evaluated within and across countries. We are the first to show that the evaluation of these protagonists systematically relates to pandemic-related behaviors and behavioral intentions. The higher a protagonist was evaluated on warmth and competence, the more participants intended to support the protagonist, and the less they intended to oppose them. Moreover, high warmth and competence evaluations of protagonists translated into pandemic-related behaviors in line with the prototypical stance of the protagonist on the matter. We hope our findings contribute to further theory development and inspire both researchers and policymakers interested in designing interventions to motivate desirable health outcomes.

## Methods

This study received ethical clearance from the institutional review board of the Department of Psychology of Durham University on 01/06/2021, and was preregistered before data analysis on 10/12/2021, https://osf.io/szc6k. All methods were performed in accordance with the relevant guidelines and regulations, including the American Psychological Association and Declaration of Helsinki. Informed consent was obtained from all participants. For online supplementary material (OSM), including complete materials, protocols, outputs, and code, see the Open Science Framework (OSF) project page, https://osf.io/3fmw2.

### Sample and procedure

We collected data from *N* = 12,553 participants living in 35 different countries. Following established stereotype content model (SCM) procedures^63^, we established a two-stage research procedure in each country, including the initial identification of relevant protagonists in the COVID-19 pandemic (pilot study; *N* = 1016) and the subsequent main survey (*N* = 11,537). Following recommendations^59^, we recruited about *N* ≥ 300 participants per country using convenience sampling for the main survey, in some cases in return for a small monetary incentive or course credit. For a list of included countries and the samples’ demographic composition for both the pilot study and main survey, see Table 4.

**Table 4:** Demographic composition for each country in the pilot study and main survey.

Country	Pilot study							Main survey						
	*n*	Collection period	Age		Gender			*n*	Collection period	Age		Gender		
			*M* (*SD*)	Range	Male (%)	Female (%)	Other (%)			*M* (*SD*)	Range	Male (%)	Female (%)	Other (%)
Argentina	21	30/06/2021–03/07/2021	33.4 (11.5)	23–67	6 (28.6)	15 (71.4)	–	273	19/08/2021–23/02/2022	27.6 (13.5)	18–81	79 (28.9)	179 (65.6)	6 (2.2)
Armenia	28	02/06/2021–14/06/2021	31.9 (9.9)	21–55	16 (57.1)	12 (42.9)	–	286	09/11/2021–17/01/2022	23.9 (8.4)	18–64	59 (20.6)	146 (51.1)	20 (7.0)
Australia	30	21/05/2021–15/06/2021	35.8 10.2)	19–58	6 (20.0)	22 (73.3)	1 (3.3)	205	26/10/2021–31/01/2022	29.6 (8.6)	18–54	38 (18.6)	166 (81.0)	1 (0.5)
Austria	25	31/05/2021–03/06/2021	27.5 (4.2)	23–38	11 (44.00)	14 (56.00)	–	311	15/09/2021–04/02/2022	27.0 (7.0)	18–60	85 (27.3)	206 (66.2)	12 (3.9)
Belgium	28	31/05/2021–01/06/2021	39.5 (11.0)	19–67	3 (10.71)	24 (85.71)	–	316	01/10/2021–30/11/2021	42.5 (9.0)	18–76	74 (23.4)	225 (71.2)	9 (2.9)
Bosnia & Herzegovina	25	28/05/2021–01/06/2021	38.4 (10.1)	23–64	9 (36.00)	16 (64.00)	–	293	07/12/2021–24/01/2022	44.0 (9.6)	18–69	103 (35.2)	164 (56.0)	3 (1.02)
Brazil	31	05/06/2021–08/06/2021	43.2 (9.9)	26–60	7 (22.58)	24 (77.42)	–	665	29/09/2021–08/03/2022	30.1 (4.3)	18–59	451 (67.8)	75 (11.3)	59 (8.9)
Bulgaria	36	11/06/2021–23/06/2021	38.0 (11.2)	25–66	6 (16.7)	26 (72.2)	–	227	09/02/2022–23/03/2022	33.8 (12.6)	18–74	70 (30.8)	139 (61.2)	3 (1.3)
Canada	38	05/07/2021–09/07/2021	20.7 (1.8)	18–27	7 (18.4)	31 (81.6)	–	366	08/09/2021–11/10/2021	20.8 (3.4)	18–45	52 (14.2)	307 (83.9)	3 (0.8)
China	24	09/08/2021–11/08/2021	28.1 (10.0)	19–48	7 (29.2)	17 (70.8)	–	255	06/11/2021–08/01/2022	28.8 (11.0)	18–57	70 (27.5)	161 (63.1)	3 (1.2)
Cyprus (GCC)	26	20/06/2021–27/06/2021	42.0 (14.5)	18–75	9 (34.6)	17 (65.4)	–	285	21/12/2021–21/02/2022	29.5 (12.2)	18–73	83 (29.1)	195 (68.4)	–
Cyprus (TCC)	37	04/10/2021–10/10/2021	37.5 (10.3)	20–41	17 (46.0)	20 (54.1)	–	295	25/11/2021–03/03/2022	35.0 (16.5)	18–85	106 (35.9)	146 (49.5)	14 (4.8)
Czech Republic	25	31/05/2021–02/06/2021	37.6 (13.1)	20–59	7 (28.0)	18 (72.0)	–	407	20/09/2021–29/11/2021	32.6 (10.0)	21–73	248 (61.0)	150 (36.9)	3 (0.7)
Finland	30	03/08/2021–26/01/2022^a^	41.5 (14.4)	23–78	6 (20.0)	22 (73.3)	1 (3.3)	293	02/02/2022–01/03/2022	37.2 (12.1)	19–74	101 (34.5)	172 (58.7)	12 (4.1)
France	36	26/05/2021–06/06/2021	34.4 (13.6)	21–65	15 (41.7)	21 (58.3)	–	287	21/09/2021–03/01/2022	40.8 (17.7)	18–81	82 (28.6)	181 (63.1)	6 (2.1)
Georgia	30	02/06/2021–04/06/2021	44.1 (9.3)	27–62	6 (20.0)	24 (80.0)	–	286	25/08/2021–14/01/2022	30.0 (12.3)	18–71	39 (13.6)	150 (52.5)	19 (6.6)
Germany	29	04/06/2021–13/06/2021	40.1 (13.9)	20–61	11 (37.9)	17 (58.6)	–	351	19/09/2021–19/10/2021	34.3 (11.2)	18–67	71 (20.2)	232 (66.1)	31 (8.8)
Ghana	32	28/05/2021–01/06/2021	33.3 (7.5)	23–52	19 (59.4)	13 (40.6)	–	309	18/11/2021–23/03/2022	22.4 (4.9)	18–60	70 (22.7)	201 (65.1)	19 (6.2)
Ireland	32	15/07/2021	40.7 (18.0)	19–72	24 (75.0)	8 (25.0)	–	535	29/09/2021–02/12/2021	23.7 (8.9)	18–72	193 (36.1)	328 (61.3)	6 (1.1)
Italy	27	24/05/2021–01/06/2021	25.3 (9.7)	20–55	10 (37.1)	16 (59.3)	1 (3.7)	290	20/09/2021–14/01/2022	28.8 (11.9)	18–86	117 (40.4)	165 (56.9)	3 (1.0)
Kazakhstan	39	24/05/2021–02/06/2021	43.5 (11.5)	18–65	18 (46.2)	21 (53.9)	–	270	27/09/2021–20/02/2022	31.1 (10.9)	18–64	72 (26.7)	125 (46.3)	19 (7.0)
New Zealand	29	18/06/2021–28/06/2021	30.6 (8.7)	18–56	13 (44.8)	16 (55.2)	–	288	17/09/2021–24/01/2022	28.1 (11.8)	18–69	93 (32.3)	187 (65.0)	5 (1.7)
Norway	48	01/06/2021–29/06/2021	40.0 (8.3)	23–72	17 (35.4)	29 (60.4)	–	238	20/09/2021–01/12/2021	28.5 (7.3)	20–71	67 (28.2)	166 (69.8)	2 (0.8)
Poland	22	18/06/2021–21/06/2021	31.3 (8.7)	20–46	6 (27.3)	16 (72.7)	–	436	16/11/2021–10/12/2021	25.6 (7.7)	18–52	56 (12.8)	372 (85.3)	5 (1.2)
Portugal	24	24/05/2021–11/06/2021	42.9 (13.7)	23–75	7 (29.2)	17 (70.8)	–	293	31/08/2021–03/01/2022	40.2 (16.3)	18–80	87 (29.7)	187 (63.8)	11 (3.8)
Romania	31	02/06/2021–12/06/2021	30.5 (13.2)	21–70	6 (19.4)	25 (80.7)	–	307	21/09/2021–30/11/2021	27.4 (10.8)	18–71	70 (22.8)	199 (64.8)	5 (1.6)
Russia	35	16/05/2021–19/05/2021	37.8 (12.0)	18–73	20 (57.1)	15 (42.9)	–	325	22/10/2021–06/12/2021	35.3 (10.0)	18–76	162 (49.9)	124 (38.2)	15 (4.6)
Slovakia	21	24/06/2021–30/06/2021	44.7 (16.4)	21–74	7 (33.3)	14 (66.7)	–	357	07/11/2021–07/12/2021	40.1 (16.9)	18–85	151 (42.3)	198 (55.5)	5 (1.4)
Spain	20	24/06/2021–14/07/2021	40.3 (12.7)	21–68	8 (40.0)	12 (60.0)	–	327	13/12/2021–16/12/2021	29.8 (9.8)	18–58	162 (49.5)	158 (48.3)	5 (1.5)
Switzerland	20	01/06/2021–18/06/2021	42.0 (12.0)	23–71	7 (35.0)	13 (65.0)	–	347	30/11/2021–06/02/2022	39.4 (16.8)	18–84	172 (49.6)	144 (41.5)	18 (5.2)
Turkey	29	15/07/2021–16/07/2021	25.1 (4.6)	19–38	7 (24.14)	22 (75.86)	–	330	28/09/2021–24/12/2021	26.1 (10.3)	18–76	90 (27.3)	220 (66.7)	8 (2.4)
UK	22	24/06/2021–10/08/2021	24.6 (4.2)	19–34	3 (13.6)	19 (86.4)	–	339	04/11/2021–21/12/2021	25.5 (11.8)	18–79	70 (20.7)	265 (78.2)	2 (0.6)
Ukraine	29	18/06/2021–28/06/2021	34.2 (11.7)	19–57	5 (17.2)	24 (82.8)	–	455	26/10/2021–01/11/2021	31.2 (10.6)	18–69	74 (16.3)	254 (55.8)	61 (13.4)
USA	33	19/06/2021–20/06/2021	33.6 (13.2)	18–70	11 (33.3)	21 (63.6)	1 (3.0)	306	30/09/2021–04/10/2021	32.5 (9.3)	18–74	167 (54.6)	129 (42.2)	8 (2.6)
Uzbekistan	24	15/05/2021–20/05/2021	31.1 (11.9)	18–67	13 (54.2)	11 (45.8)	–	384	30/09/2021–21/12/2021	23.1 (8.8)	18–72	62 (16.2)	212 (55.2)	16 (4.2)

Percentages may not add up to 100% for gender due to the exclusion of missings or ‘prefer not to say’ responses. Data were collected online in all countries, with the exception of the main survey in Turkey, where we partially used paper-and-pencil questionnaires.

^a^Data collection was paused from 13/08/2021 to 19/01/2022.

Data collection periods varied between countries (15/05/2021–26/01/2022 for the pilot study, 19/08/2021–23/03/2022 for the main survey). Most data were collected using online surveys with sporadic paper–pencil surveys. All study materials were translated into other languages following a parallel translation procedure (TRAPD)^64^.

In the pilot study, we asked participants to nominate “as many individuals, groups of people, organizations and movements as [they] can when [they] think of the COVID-19 pandemic in [Country]” in an open-ended question format (see OSM-6 on OSF-page for complete questionnaires). These nominations were summarized into distinct, specific categories and rank-ordered according to frequency of nomination. Reviewing the relevant protagonists in the different countries, we identified three key protagonists who were nominated in almost all countries: (I) the heads of state; (II) physicians; (III) protest movements. We included these three protagonists in the main survey in all countries to investigate cross-country differences in the social perception of these protagonists and links to general and protagonist-specific pandemic-related behaviors. Additionally, nine protagonists from the individual country nominations were included in the main survey based on their nomination frequency, distinctiveness, and societal relevance.

The main survey included the social perception (i.e., warmth and competence assessments) of the twelve protagonists, various COVID-19-related behavioral intentions, and further variables not relevant to this study (see OSM-6).

### Measures

#### Social perception

Based on the SCM^20^, we used perceived warmth and competence scales as measures for social perception. Many SCM scales have been proposed, with some items generally performing better than others, and some being only very narrowly applicable to certain protagonists (e.g., individuals, social groups, or organizations^59^). Thus, based on their performance in previous studies^59^ and expert discussions with leading SCM researchers in the field, we proposed six traits each assessing warmth (e.g., *good-natured, cooperative*) and competence (e.g., *capable, competent*) which are equally applicable to different types of protagonists. Both scales used a 5-point Likert scale. In line with previous research^20^, we asked participants for society’s rather than their personal perspective on protagonists. This operationalization has been shown to produce equivalent results in samples of different compositions (e.g., student and general population samples) within the same country context^20^, which aligned well with our convenience sampling strategy (see^65^, for a discussion).

#### Pandemic-related behaviors

We measured three different facets of pandemic-related behaviors: Support and opposition behavior (intentions) towards the key protagonists ‘heads of state’, ‘physicians’, and ‘protest movements’; compliance with general COVID-19 prevention measures; and vaccination behavior. Behavior (intentions) towards the three key protagonists were assessed using a support (five items, e.g., *supporting the actions of [key protagonist] with relation to the COVID-19 pandemic*) and an opposition subscale (three items, e.g., *having actively defied the instructions given by [key protagonist] regarding the COVID-19 pandemic*) answered on a 5-point Likert scale.

To assess compliance with general COVID-19 prevention measures, we used a list of up to eleven behaviors (e.g., keeping distance, using pandemic-related smartphone applications, adhering to curfews) adapted from the GESIS Panel^66^. The scale was adapted to country-specific conditions by excluding behaviors that were not applicable to countries (e.g., adhering to curfews was dropped if there were no curfews). We asked participants to indicate which behaviors they have shown in the past six months and computed a relative score of prevention measures that participants adhered to (ranging from 0 *adhered to none*, to 1 *adhered completely*).

To assess vaccination behavior, we asked participants if they had already received a vaccination against COVID-19 (yes/no), and if so, how many doses they had received and how many doses are required for full effectiveness of the vaccine (1/2/ > 2). We combined responses into one vaccination behavior indicator with three levels: Not vaccinated (0), partially vaccinated (1), and fully vaccinated (2).

### Analytical strategy

We used IBM-SPSS (Version 27), Mplus (Version 8.5 or above) and R (Version 4.04.5 or higher, packages MplusAutomation, openxlsx, rstudioapi) for data analysis. We excluded participants from analysis who did not live in the country of data collection during the COVID-19 pandemic or who indicated not to have answered truthfully. Participants could skip questions they did not want to answer. We used robust full-information maximum likelihood estimators (MLR) in all quantitative analyses, which is a recommended procedure to estimate missing values based on the observed variance covariance matrix^67^.

To identify relevant protagonists who were salient in the COVID-19 pandemic, we used content analysis to summarize and quantify the different relevant protagonists across all countries^20^. For the remaining analyses, as an initial step, we conducted confirmatory factor analysis (CFA) for the multi-item scales warmth, competence, supportive and opposing behavior (intentions) to ensure adequate scale reliability and dimensionality. We fitted and adapted the CFAs separately for each country, resulting in scales that are not completely comparable in meaning (i.e., emic concepts^68^). Nonetheless, following previous research^65^, the warmth and competence scales were kept identical for all protagonists *within* each country when investigating how protagonists were perceived *within* countries and identical for each key protagonist *across* all countries when investigating how protagonists are perceived *across* countries to ensure equivalence of meaning for each comparison. To identify the best measurement model for each set of analysis, we developed an automation tool in R, which we describe and provide in more detail in OSM-2. In brief, the tool was tasked to find the best fitting and most parsimonious measurement model within each set of analyses (i.e., the ideal solution for all the protagonists *within* countries, or identical key protagonists *across* countries) based on pre-defined parameters we describe in the analysis plan document in OSM-1. We excluded protagonists from the respective analysis if we could not establish an adequately fitting CFA model. To compare the protagonists’ social perception within and across countries for each key protagonist, we ran fixed alignment optimization procedures^45^, which generate a mathematically optimized measurement invariance pattern to ensure meaningful comparability of the latent mean scores for warmth and competence for each protagonist. To investigate how social perceptions of the key protagonists relate to pandemic-related behavior (intentions), we fit a series of models for each country and key protagonist correlating warmth and competence with the different pandemic-related behaviors (intentions). We optimized the measurement models for the behavior intention measures for each key protagonist in each country separately using the same criteria as for SCM measures. The separate correlation coefficients were summarized using meta-analysis^69^ to examine whether warmth and competence related to pandemic-related behavior (intentions) in the expected way. Details on analytical procedures are reported in OSM-1.

## Data Availability

The analyses for this article were preregistered on 10th Dec 2021 at the Open Science Framework, see https://osf.io/szc6k. All materials, data and analysis code are provided on the article’s Open Science Framework project page, see https://osf.io/3fmw2/. A comprehensive overview of the findings of this article was published in the ShinyApp https://jaherzig.shinyapps.io/COVID19-protagonists/.
