# Author Correction: Warmth and competence perceptions of key protagonists are associated with containment measures during the COVID-19 pandemic: Evidence from 35 countries

**DOI:** 10.1038/s41598-023-27832-9

**Published:** 2023-01-12

**Authors:** Maria-Therese Friehs, Patrick F. Kotzur, Christine Kraus, Moritz Schemmerling, Jessica A. Herzig, Adrian Stanciu, Sebastian Dilly, Lisa Hellert, Doreen Hübner, Anja Rückwardt, Veruschka Ulizcay, Oliver Christ, Marco Brambilla, Jonas De keersmaecker, Federica Durante, Jessica Gale, Dmitry Grigoryev, Eric R. Igou, Nino Javakhishvili, Doris Kienmoser, Gandalf Nicolas, Julian Oldmeadow, Odile Rohmer, Bjørn Sætrevik, Julien Barbedor, Franco Bastias, Sebastian B. Bjørkheim, Aidos Bolatov, Nazire Duran, Andrej Findor, Friedrich Götz, Sylvie Graf, Anna Hakobjanyan, Georgios Halkias, Camellia Hancheva, Martina Hřebíčková, Matej Hruška, Shenel Husnu, Kamoliddin Kadirov, Narine Khachatryan, Francisco G. Macedo, Ana Makashvili, Maylin Martínez-Muñoz, Eric Mercadante, Luiza Mesesan Schmitz, Andreas Michael, Nozima Mullabaeva, Félix Neto, Joana Neto, Merve Ozturk, Svitlana Paschenko, Agnieszka Pietraszkiewicz, Charis Psaltis, Yuting Qiu, Mirjana Rupar, Adil Samekin, Katharina Schmid, Sabine Sczesny, Yiwen Sun, Annika M. Svedholm-Häkkinen, Aleksandra Szymkow, Enoch Teye-Kwadjo, Claudio V. Torres, Luc Vieira, Illia Yahiiaiev, Vincent Yzerbyt

**Affiliations:** 1 FernUniversität in Hagen, Hagen, Germany; 2 Department of Psychology, Durham University, South Road, Durham, DH1 3LE UK; 3 GESIS Leibniz-Institut für Sozialwissenschaften, Mannheim, Germany; 4 University of Milano-Bicocca, Milan, Italy; 5 Esade, Ramon Llull University, Barcelona, Spain; 6 University of Canterbury, Christchurch, New Zealand; 7 HSE University, Moscow, Russia; 8 University of Limerick, Limerick, Ireland; 9 Ilia State University, Tbilisi, Georgia; 10 Rutgers University, New Brunswick, USA; 11 Swinburne University of Technology, Melbourne, Australia; 12 University of Strasbourg, Strasbourg, France; 13 University of Bergen, Bergen, Norway; 14 Université Catholique de Louvain, Louvain-la-Neuve, Belgium; 15 Universidad Católica de Cuyo/National Scientific and Technical Research Council, San Juan, Argentina; 16 Astana Medical University, Astana, Kazakhstan; 17 Comenius University in Bratislava, Bratislava, Slovakia; 18 The University of British Columbia, Vancouver, Canada; 19 The Czech Academy of Sciences, Prague, Czechia; 20 Yerevan State University, Yerevan, Armenia; 21 Copenhagen Business School, Frederiksberg, Denmark; 22 Sofia University “St. Kliment Ohridski”, Sofia, Bulgaria; 23 Eastern Mediterranean University, Famagusta, Cyprus; 24 University of Innovative and Social Economics, Tashkent, Uzbekistan; 25 University of Brasilia, Brasília, Brazil; 26 Transilvania University of Brasov, Brașov, Romania; 27 University of Cyprus, Nicosia, Cyprus; 28 National University of Uzbekistan, Tashkent, Uzbekistan; 29 University of Porto, Porto, Portugal; 30 Universidade Portucalense, Porto, Portugal; 31 Taras Shevchenko National University of Kyiv, Kyiv, Ukraine; 32 University of Bern, Bern, Switzerland; 33 M. Narikbayev KAZGUU University, Astana, Kazakhstan; 34 Tampere University, Tampere, Finland; 35 SWPS University of Social Sciences and Humanities, Warsaw, Poland; 36 University of Ghana, Accra, Ghana; 37 Jagiellonian University, Krakow, Poland

Correction to: *Scientific Reports* 10.1038/s41598-022-25228-9, published online 08 December 2022

The original version of this Article contained an error in Affiliation 27, which was incorrectly given as ‘University of Nicosia, Nicosia, Cyprus’. The correct Affiliation is listed below:

University of Cyprus, Nicosia, Cyprus.

The original Article has been corrected.

